# GLP-1 and Its Derived Peptides Mediate Pain Relief Through Direct TRPV1 Inhibition Without Affecting Thermoregulation

**DOI:** 10.21203/rs.3.rs-4233732/v1

**Published:** 2024-05-13

**Authors:** Chul-Kyu Park, Eun Jin Go, Hyunjung Jo, Sung-Min Hwang, Md. Mahbubur Rahman, Jaeik Park, Ji Yeon Lee, Youn Yi Jo, YunJae Jung, Temugin Berta, Yong Ho Kim

**Affiliations:** Gachon University; Gachon University; Gachon University; Gachon University; Gachon University; Gachon University; Gil Medical Center, Gachon University; Gil Medical Center, Gachon University; Gachon University; University of Cincinnati Medical Center; Gachon University

## Abstract

Hormonal regulation during food ingestion and its association with pain prompted the investigation of the impact of glucagon-like peptide-1 (GLP-1) on the transient receptor potential vanilloid 1 (TRPV1). Both endogenous and synthetic GLP-1 and an antagonist of GLP-1, exendin 9–39, reduced heat sensitivity in naïve mice. GLP-1-derived peptides (liraglutide, exendin-4, and exendin 9–39) effectively inhibited capsaicin (CAP)-induced currents and calcium responses in cultured sensory neurons and TRPV1-expressing cell lines. Notably, the exendin 9–39 alleviated CAP-induced acute pain, as well as chronic pain induced by complete Freund’s adjuvant (CFA) and spared nerve injury (SNI) in mice, without causing hyperthermia associated with other TRPV1 inhibitors. Electrophysiological analyses revealed that exendin 9–39 binds to the extracellular side of TRPV1, functioning as a noncompetitive inhibitor of CAP. Exendin 9–39 did not affect proton-induced TRPV1 activation, suggesting its selective antagonism. Among exendin 9–39 fragments, exendin 20–29 specifically binds to TRPV1, alleviating pain in both acute and chronic pain models without interfering with GLP-1R function. Our study revealed a novel role for GLP-1 and its derivatives in pain relief, proposing exendin 20–29 as a promising therapeutic candidate.

## INTRODUCTION

Individuals experiencing pain often report overeating calorie-dense, high-sugar, and high-fat foods^1^ as a coping mechanism^2^. This phenomenon, termed “ingestion analgesia,” has been also observed in animals^3–6^. Noxious heat-evoked withdrawal behaviors in rats are suppressed during self-initiated chocolate eating and ingestion of sucrose^7^. While much research has focused on mechanisms within the central nervous system to explain this pain suppression^8^, we hypothesize additional mechanisms occurring in the peripheral nervous system based on hormonal regulation during digestion.

Upon food ingestion and its subsequent entry into the small intestine, intestinal L-cells undergo posttranslational processing of the proglucagon gene, leading to the production of glucagon-like peptide-1 (GLP-1), a potent incretin peptide hormone^9^. GLP-1 plays an important role in glucose homeostasis and is secreted into the hepatic portal system in response to elevated glucose levels from food intake^10^, stimulating insulin synthesis and release from the pancreas^11^. The short half-life of native GLP-1, typically only 1–2 min due to rapid degradation, has facilitated the development of structurally modified GLP-1 analogs with longer half-lifes, such as liraglutide, exendin-4, and dulaglutide, which exhibit 97%, 53%, and 90% sequence homology, respectively^12^.

Regarding pain modulation, various studies have reported that GLP-1 analogs exert antinociceptive effects on the central nervous system. This modulation occurs by regulating the spinal dorsal horn microglial pathway through GLP-1R activation, leading to alterations in spinal excitatory synaptic transmission under neuropathic pain conditions^13–18^. However, the effects of GLP-1 on the peripheral nervous system, and its relationship with pain modulation, remain largely unexplored.

The transient receptor potential vanilloid 1 (TRPV1) channel plays a crucial role in heat pain perception^19^. TRPV1, known for its thermosensitive and polymodal nociceptor properties, is expressed in sensory neurons and is activated by stimuli such as capsaicin (CAP) and protons^20^. In chronic pain states, TRPV1 channels are upregulated in nociceptive neurons, which lowers stimulation thresholds and increases pain perception, as reported in hyperalgesia or allodynia^21^. Inhibiting TRPV1 has proven effective in mitigating pain in diverse neuropathic pain models, thereby garnering interest from numerous pharmaceutical companies^22,23^. However, the challenge with current TRPV1 antagonists lies in their associated adverse effects, such as hyperthermia^24–31^ and hypothermia^32–34^, caused by failures in thermoregulation. This issue has been attributed to the mode of action of these antagonists: those blocking CAP-, proton-, and heat-induced TRPV1 activation result in hyperthermia, while those sparing proton-induced activation do not lead to hyperthermia^33,35^. Conversely, potentiation of proton-induced TRPV1 activation leads to hypothermia^32^.

In light of these challenges, in this study, we assess the impact of GLP-1 and its derivatives on pain behavior and their influence on the peripheral nervous system. Preliminary findings indicate that GLP-1 harbors a key sequence that directly binds to and inhibits the activation of TRPV1 channels in sensory neurons. Importantly, we also demonstrate that GLP-1 and its derivatives directly anatgonize TRPV1 channels in a mode-selective manner that can offer pain relief without the adverse thermoregulatory side effects commonly associated with current TRPV1 inhibitors.

## MATERIALS AND METHODS

### Chemicals

CAP and N-(4-tertiarybutylphenyl)-4-(3-cholorphyridin-2-yl) tetrahydropyrazine-1(2H)-carboxamide (BCTC) were purchased from Sigma-Aldrich (St. Louis, MO), and the stock solutions were prepared with 99.5% ethanol and dimethyl sulfoxide, respectively. AITC and CFA was purchased from Sigma-Aldrich. GLP-1(7–36), liraglutide, exendin-4, exendin 9–39, exendin 20–29, His-tagged exendin 9–39, and FITC-tagged exendin 9–39 were purchased from Anygen Corp. (Gwangju, South Korea). The stock solutions were prepared according to the supplier’s recommendation for each peptide and were stored at − 20°C. The exendin 20–29 alanine scanning library was purchased from Genscript (Piscataway, NJ).

### Animals

Adult wild-type male C57BL/6N mice were purchased from Orient Bio (Sungnam, South Korea). Mice were group-housed at constant temperature and humidity under a 12-h/12-h light-dark cycle with free access to standard food and water for at least 1 week prior to the beginning of the experimental procedures. All animal experiments were approved by the Institutional Animal Care and Use Committee of the College of Medicine at Gachon University (approval number: LCDI-2020–0135).

### Cell preparation and transient transfection

DRGs were aseptically removed from 5–8-week-old mice and were incubated with collagenase A (0.2 mg/mL; Roche, Basel, Switzerland)/dispase-II (2.4 units/mL, Roche) at 37°C for 90 min. Cells were mechanically dissociated by gentle pipetting and placed on glass coverslips coated with poly-D-lysine.

DRG cells were then grown in a neurobasal-defined medium supplemented with 10% fetal bovine serum (FBS; Gibco, Waltham, MA), 2% B27 supplement (Invitrogen, Carlsbad, CA), and 1% penicillin/streptomycin for 24 h before experiments. Human TRPV1 (hTRPV1)-expressing CHO K1 cells were cultured using Dulbecco’s modified Eagle’s medium (DMEM) containing 10% FBS, 1% penicillin/streptomycin, and 800 μg/mL geneticin in an incubator with 5% CO_2_ at 37°C. HEK293 and HEK293T cells were also cultured in the same DMEM but without geneticin. Rat TRPV1 plasmids were labeled with a green fluorescent protein to indicate subsequent calcium imaging. cDNA constructs of rat TRPV1 were transiently transfected into cells using Lipofectamine 2000 (Invitrogen). Ca^2+^ imaging was performed one day after transient transfection.

### Patch-clamp recordings

Whole-cell voltage-clamp and cell-attached patch-clamp recordings were conducted at room temperature to measure capsaicin (CAP)- or proton-induced currents in dissociated dorsal root ganglion (DRG) neurons and hTRPV1-expressing CHO K1 cells, respectively. An EPC10 amplifier (HEKA, Stuttgart, Germany) was utilized for these recordings. Patch pipettes, prepared with a micropipette puller (Narishige, Tokyo, Japan), had resistances of 4–5 MΩ for whole-cell recordings and 6–8 MΩ for cell-attached recordings. The recording chamber, with a volume of 500 μL, was continuously superfused at a rate of 1–2 mL/min. CAP- or low pH-induced currents were recorded at a holding potential of − 60 mV. Series resistance was compensated (> 80%), and leak subtraction was performed. The data acquired were low-pass filtered at 2 kHz and sampled at 10 kHz, analyzed using PatchMaster and FitMaster (HEKA) software. The internal solution for whole-cell and cell-attached recordings consisted of 126 mM K-gluconate, 10 mM NaCl, 1 mM MgCl_2_, 10 mM EGTA, 10 mM HEPES, 2 mM NaATP, and 0.1 mM Na_2_GTP, adjusted to pH 7.3 with KOH, osmolarity 295–300 mOsm. The external solution of 140 mM NaCl, 5 mM KCl, 1 mM MgCl_2_, 2 mM EGTA, 10 mM HEPES, and 10 mM glucose, adjusted to pH 7.4 with NaOH, osmolarity 300–310 mOsm, was used for both techniques.

Inside-out recordings from hTRPV1-expressing CHO K1 cells were conducted using a pipette resistance of 8–9 MΩ. The internal solution for inside-out recordings matched that used for whole-cell and cell-attached methods, whereas the external solution (intracellular side) differed slightly, containing 2 mM CaCl_2_ instead of EGTA. The open probability and average single-channel opening and closing times for inside-out recordings were analyzed using a 50% threshold criterion, with all events double-checked before analysis.

### Ca^2+^ imaging in dissociated DRG neurons and hTRPV1-CHO K1 cells

At room temperature, Ca^2+^ imaging was conducted in mouse DRG, HEK293T, and hTRPV1-expressing CHO K1 cells. Cells on poly-D-lysine-coated coverslips were loaded with 2 μM Fura-2 AM (Thermo Fisher Scientific, Waltham, MA) at 37°C for 40 min in DMEM. The cells were then rinsed three times with the medium and incubated for 30 min, following which they were placed on the stage of an inverted microscope (BX51W1; Olympus, Tokyo, Japan) and continuously superfused at a flow speed of 1 mL/min with a bath solution containing 140 mM NaCl, 5 mM KCl, 1 mM CaCl_2_, 2 mM MgCl_2_, 10 mM HEPES, and 10 mM glucose, adjusted to pH 7.4 with NaOH. Using illumination with a 175-W xenon arc lamp, excitation wavelengths (340/380 nm) were selected using a Lambda DG-4 monochromator wavelength changer (Shutter Instrument, Novato, CA). The fluorescence 340/380 ratio was measured using digital video microfluorometry with an intensified camera (OptiMOS; QImaging, Surrey, Canada) coupled to the microscope. Data were analyzed using SlideBook 6 (Intelligent Imaging Innovations, Denver, CO). At the end of the experiment, cells were identified based on their response to high concentrations of KCl.

### Pull-down assay and immunoblotting

Interactions between exendin 9–39 and TRPV1 were examined via His-mediated pull-down assays using a modified protocol from the Pierce Pull-down PolyHis Protein:Protein Interaction Kit (Thermo Fisher Scientific). His-tagged exendin 9–39 was bound to HisPur Cobalt Resin as the bait protein and incubated with lysates of hTRPV1-CHO K1 or native CHO K1 cells overnight at 4°C. After washing five times with lysis buffer, the complexes were mixed with lysis buffer containing 290 mM imidazole, and the bound proteins were eluted by boiling in 5× sodium dodecyl sulfate (SDS) loading buffer for 5 min.

The products were then separated using SDS-polyacrylamide gel electrophoresis, transferred onto nitrocellulose membranes, and blotted with an anti-TRPV1 antibody (#ACC-030; Alomone Labs, Jerusalem, Israel). The membrane was washed with TBST and incubated for 1 h with the secondary antibody, anti-rabbit Ig-HRP (1:10,000, 9910; Cell Signaling Technologies, Danvers, MA). After washing with TBST, the immune complexes were detected by chemiluminescence (Beyotime, Shanghai, China) using a Pierce western blotting kit (Thermo Fisher Scientific). Quantitative densitometric analysis was performed using a UVP BioSpectrum multispectral imaging system (Image Quant LAS 4000; GE Healthcare, Chicago, IL).

### Confocal fluorescence imaging

Approximately 1 × 10^5^ mL^−1^ of hTRPV1-expressing CHO K1 cells or naïve CHO K1 cells were seeded into confocal dishes and cultured overnight for cell adherence. The cells were washed with phosphate-buffered saline (PBS) and fixed with 2% paraformaldehyde for 10 min at 25°C. After washing with PBS, the cells were permeabilized with 0.1% Triton X-100 for 5 min at room temperature and blocked with 3% bovine serum albumin with glycine for 30 min. Cells were incubated with primary antibody against TRPV1 (1:250; #ACC-030, Alomone Labs) for 1 h at room temperature. After washing with PBS three times, Alexa Fluor 594-conjugated secondary antibody (1:400; Invitrogen) and Hoechst 33342 (Thermo Fisher Scientific) were added for another hour of incubation at 4°C. The cells were washed with PBS and incubated with FITC-tagged exendin 9–39 dissolved in cold PBS at a concentration of 10 μM for 30 min at 4°C. Finally, after washing with cold PBS, images were acquired using a confocal laser-scanning microscope with a 100× oil-immersion objective (LSM 700; Carl Zeiss, Oberkochen, Germany).

### Behavioral tests in mice

Baseline heat sensitivity was measured to determine the systemic effect of glucose or GLP-1(7–36) administration using a hot plate (Ugo Basile, Italy). The PWL to heat was measured after intraperitoneal administration of glucose (2.0 g/kg body weight) in a volume of 200 μL or after administration of the same volume of vehicle (0.9% saline) (*n* = 5 each). The local effect of GLP-1(7–36) and exendin 9–39 in heat sensitivity was tested by measuring PWL to heat using the Hargreaves radiant heat apparatus after intraplantar administration of 10 μg GLP-1(7–36) or vehicle (0.9% saline) (n = 5 each) in a volume of 10 μL.

To evaluate nociceptive behavior, the time spent licking the paw, the paw withdrawal threshold (PWT) to mechanical stimuli, and the PWL to heat were measured as described previously^36^. Either vehicle, exendin 9–39, exendin 20–29, or BCTC was first injected via the intraplantar route, and 30 min later, an additional exendin 9–39, exendin 20–29, or BCTC in combination with CAP was injected.

Licking time was recorded for 5 min after CAP injection in each group. Mechanical allodynia and thermal hyperalgesia were evaluated in separate experiments in a time-dependent manner. Mechanical allodynia was assessed using von Frey filaments (NC12775–99; North Coast Medical, CA). The 50% PWT was calculated using the up-down method. Thermal hyperalgesia was assessed by recording PWL using the Hargreaves radiant heat apparatus (IITC Life Sciences, Woodland Hills, CA). A cutoff value of 20 s was used to prevent tissue damage. For rectal temperature recording, the rectal temperature was measured with a digital thermometer (Therma-1; ETI, West Sussex, UK) by inserting a corn oil-soaked flexible bead probe into the rectum after intraperitoneal administration of 200 μL of vehicle, exendin 9–39 (50 μg/kg), or BCTC 5 mg/kg in wild-type mice (*n* = 6). The acute IPGTT was performed in mice aged 8 weeks. Baseline blood glucose levels were measured and mice were intraperitoneally injected with 200 μL of vehicle, exendin 20–29 (10 μg/kg), and exendin-4 (10 μg/kg) in wild-type mice (n = 6). After 15 min, the mice were challenged with glucose (2.0 g/kg body weight). Blood glucose levels were measured using an Accu-Chek Performa glucometer (Roche, Mannheim, Germany).

#### CFA-induced pain

Under temporal anesthesia with 3% isoflurane, the CFA-induced inflammatory pain model was established by intraplantar injection of 20 μL of CFA. Mice were then treated with either vehicle, exendin 9–39, or exendin 20–29 in 20 μL, administered on the same paw where CFA was injected. Heat hyperalgesia and mechanical allodynia induced by CFA were assessed using the Hargreaves and von Frey tests, respectively. Paw thickness was measured in millimeters using a digimatic caliper CD-15APX (Mitutoyo Corporation, Kawasaki, Japan).

#### SNI-induced pain

Under continuous anesthesia with isoflurane, mice underwent surgical manipulation to expose the left sciatic nerve by separating the muscle tissue. Upon visualization of the sciatic nerve, the peroneal and tibial nerves were ligated and transected at the lower end of the ligature using silk thread, while the sural nerve remained intact. The surgical site was then sutured, and iodine was applied for debridement. After a recovery period of 14 days post-surgery, mice received either intraplantar or intraperitoneal administration (20 or 200 μL, respectively) of vehicle, exendin 9–39, or exendin 20–29. Subsequently, the heat sensitivity of the neuropathic pain model mice was assessed using the Hargreaves test.

#### Statistical analysis

Statistical analyses were conducted using GraphPad Prism 8 (GraphPad Software, San Diego, CA). All data are presented as the mean ± standard error of the mean (S.E.M.). Differences between groups were compared using a two-tailed unpaired t-test for two groups, one-way analysis of variance (ANOVA) followed by Dunnett’s multiple comparison test for multiple groups, or two-way repeated measures ANOVA followed by the Bonferroni multiple comparison test for multiple groups and time courses. The statistical significance thresholds were * *p* < 0.05, ** *p* < 0.01, *** *p* < .001, and **** *p* < .0001 (likewise indicated with # and †).

## RESULTS

### Release of GLP-1 by glucose application alleviates heat sensitivity

**Figure 1:**
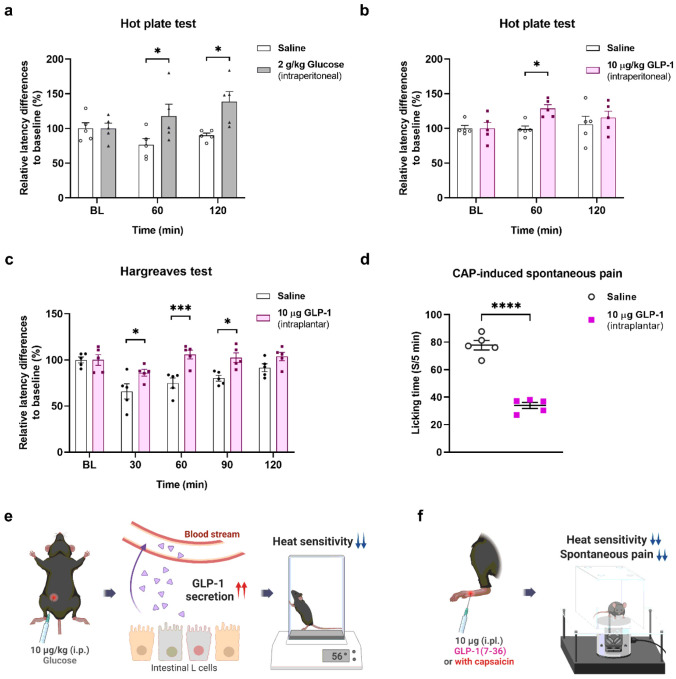
Reduced thermal sensitivity in systemically glucose or GLP-1 treated- and locally GLP-1 treated-mice and analgesic effect of GLP-1 on CAP-induced nociceptive behaviors in mice. **a** Effects of intraperitoneal administration (i.p.) of 2 g/kg glucose on heat sensitivity using hot plate test (mean ± S.E.M., *n* = 5). Two-way ANOVA followed by Bonferroni multiple comparison test (*p < 0.05, compared with the saline group). **b** Effects of intraperitoneal administration of 10 μg/kg GLP-1(7–36) (GLP-1) on heat sensitivity using hot plate test (mean ± S.E.M., *n*= 5). Two-way ANOVA followed by Bonferroni multiple comparison test (*p< 0.05, compared with the saline group). **c** Effects of intraplantar administration (i.pl.) of 10 μg GLP-1(7–36) on heat sensitivity using the Hargreaves test (mean ± S.E.M., *n* = 5). Two-way ANOVA followed by Bonferroni multiple comparison test (*p < 0.05, ***p <.001, compared with the saline group). **d** Effects of intraplantar administration of 10 μg of GLP-1(7–36) on CAP (1.6 μg)-induced spontaneous licking time (mean ± S.E.M., *n* = 5). Two-tailed unpaired t-test (****p<.0001, compared with the saline group). Schematic illustration of the analgesic effect through systemic GLP-1 secretion (**e**) and local GLP-1 induction (**f**). ANOVA: analysis of variance, CAP: capsaicin, GLP-1: glucagon-like peptide-1, S.E.M: standard error of mean.

Given the essential role that GLP-1 plays in regulating glucose levels in the body, we primarily utilized intraperitoneal glucose administration and assessed changes in heat sensitivity in mice using hot plate tests. The role of endogenous GLP-1 in relation to heat sensitivity was demonstrated by the observation that the intraperitoneal glucose-treated group exhibited a decrease in heat sensitivity as compared with the vehicle-treated group (Fig. 1a), as the administration of glucose significantly increased blood glucose levels from 1 to 2 h (Supplementary Fig. 1). To confirm whether the reduction in heat sensitivity after glucose application was due to systemic release of GLP-1, we intraperitoneally administered 10 μg/kg GLP-1(7–36), one of the two primary biologically active forms of secreted GLP-1. Similar to the results observed with glucose administration, the hot plate test revealed a reduction in heat sensitivity from 1 h (Fig. 1b, e).

Upon discovering the impact of systemic GLP-1 on heat sensitivity, we evaluated its influence on the peripheral nervous system through local intraplantar injection of 10 μg GLP-1(7–36). The results of the Hargreaves test demonstrated a reduction in pain sensitivity (Fig. 1c). As changes in heat sensitivity are dependent on TRPV1 activity, we sought to determine whether intraplantar injection of 10 μg GLP-1(7–36) modulates TRPV1 agonist CAP-induced spontaneous pain behavior in mice. We found that intraplantar injection of 10 μg GLP-1(7–36) for 10 min significantly reduced pain-like (licking) behavior induced by intraplantar injection of 1.6 μg CAP (Fig. 1d). Therefore, we hypothesized that GLP-1 and its metabolites affect peripheral pain regulation through the TRPV1 channel (Fig. 1f).

### GLP-1 and its analogs modulate CAP-induced TRPV1 activation in mouse dorsal root ganglia (DRG) neurons

**Figure 2:**
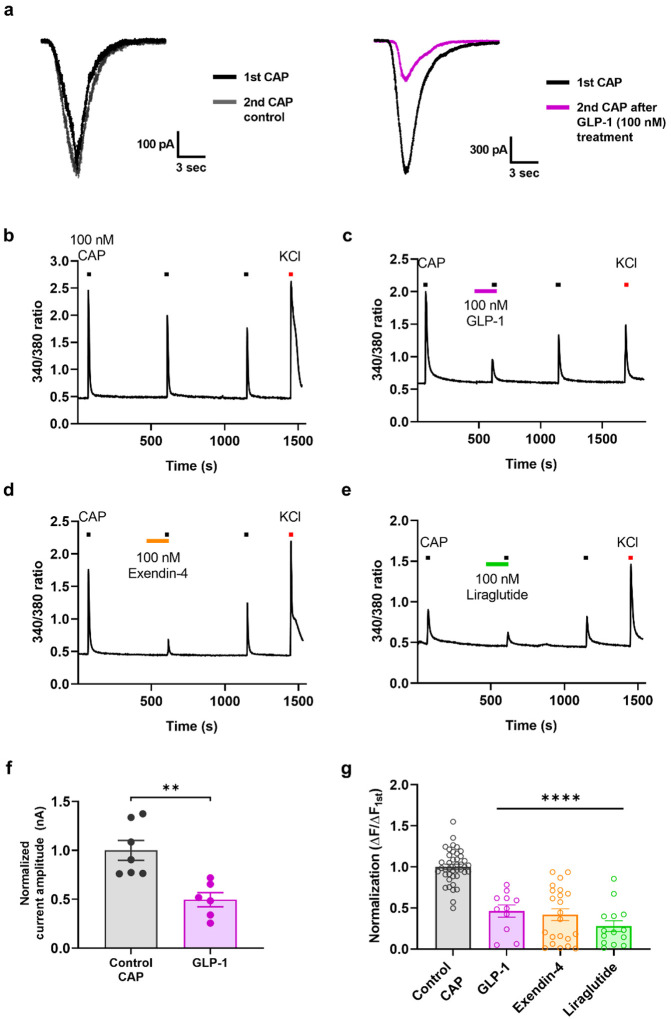
Inhibition of CAP-induced TRPV1 currents and calcium responses in small-to-medium-sized mouse DRG neurons by pretreatment with GLP-1 analogs. **a**Representative inward currents induced by 100 nM CAP in the absence (left) or presence of 100 nM GLP-1(7–36) (GLP-1; right). **b–e** Representative traces of calcium influx elicited by 100 nM control CAP (**b**), GLP-1 (**c**), exendin-4 (d), or liraglutide (**e**). The calcium response to high potassium (KCl, 50 mM) was used to identify neurons. **f** Mean normalized currents of sequential CAP-induced currents (mean ± S.E.M.). Two-tailed unpaired *t*-test (**p < 0.01, compared with each control CAP). **g** Mean normalized 340/380 ratios of sequential CAP-induced calcium increases (mean ± S.E.M.). One-way ANOVA followed by Dunnett’s multiple comparison test (****p<.0001, compared with control CAP). ANOVA: analysis of variance, CAP: capsaicin, DRG: dorsal root ganglia, GLP-1: glucagon-like peptide-1, S.E.M: standard error of mean, TRPV1: transient receptor potential vanilloid 1.

To determine whether GLP-1 regulates peripheral pain signaling by modulating TRPV1 activity in sensory neurons, whole-cell patch-clamp recordings were performed using small-diameter (< 25 μm) neurons dissociated from mouse DRG. The application of 100 nM CAP elicited inward currents, indicating CAP-responsive DRG neurons, and these responses were abolished by pretreatment with 100 nM GLP-1(7–36) by 52%. (Fig. 2a, f). We further tested TRPV1 channel modulation using Ca^2+^ imaging (Fig. 2b–e, g). Pretreatment with each examined GLP-1 analogs, including GLP-1(7–36), liraglutide, and exendin-4, led to significantly reduced Ca^2+^ responses (by 53.8%, 58.1%, and 72.2%, respectively). These results suggest that GLP-1 analogs play an antagonistic role in regulating TRPV1 function.

### The antagonist of GLP-1, exendin 9–39, regulates nociception via TRPV1 modulation

Exendin 9–39, a synthetic peptide, functions as a specific and competitive antagonist of GLP-1^37^. It is derived from exendin-4 through N-terminal truncation and shares 53% sequence homology with native GLP-1^38^.

**Figure 3:**
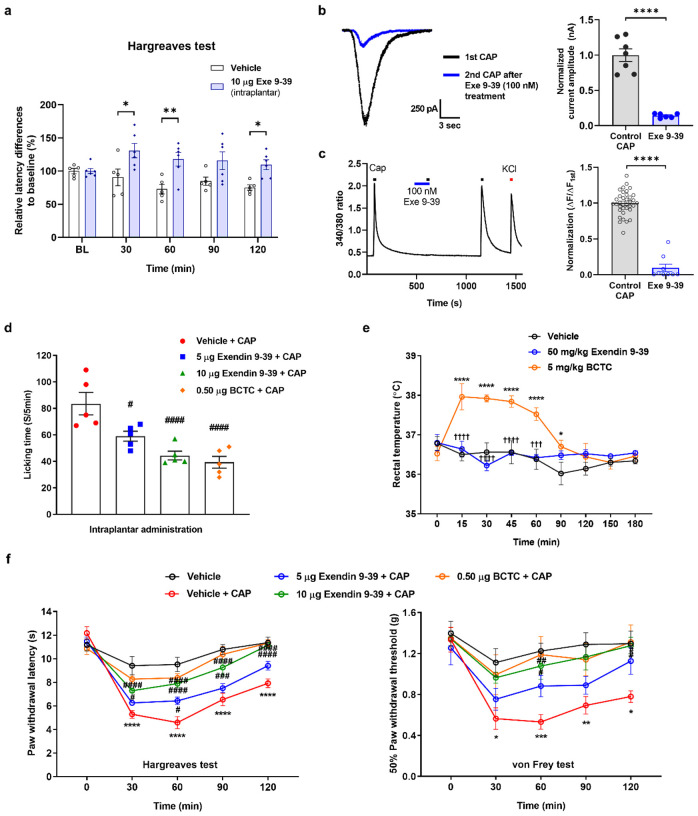
Inhibition of CAP-induced TRPV1 currents and calcium responses in small-to-medium-sized mouse DRG neurons by pretreatment with exendin 9–39, an antagonist of GLP-1, and its analgesic effects on CAP-induced nociceptive behaviors in mice. **a**Effects of intraplantar administration of exendin 9–39 (Exe 9–39) (10 μg) on heat sensitivity using the Hargreaves test. Two-way ANOVA followed by Bonferroni multiple comparison test (*p < 0.05, compared with the saline group). **b** Representative inward currents induced by 100 nM CAP in the presence of 100 nM exendin 9–39 (Exe 9–39; left). Mean normalized currents of sequential CAP-induced currents (mean ± S.E.M.; right). Two-tailed unpaired *t*-test (****p <.0001, compared with each control CAP). cRepresentative traces of calcium influx elicited by 100 nM control CAP and pretreatment of 100 nM Exe 9–39. The calcium response to high potassium (KCl, 50 mM) was used to identify neurons (left). Mean normalized 340/380 ratios of sequential CAP-induced calcium increases (mean ± S.E.M.; right). Two-tailed unpaired *t*-test (****p <.0001, compared with each control CAP). **d** Effects of intraplantar administration of exendin 9–39 (5 and 10 μg) and BCTC (0.5 μg) on CAP (1.6 μg)-induced acute licking time (mean ± S.E.M., *n* = 5). One-way ANOVA followed by Dunnett’s multiple comparison test (#*p* < 0.05, ####*p* <.0001, compared with vehicle + CAP group). **e** Effects of intraperitoneal administration of exendin 9–39 (50 mg/kg) and BCTC (5 mg/kg) on body temperature (mean ± S.E.M., *n* = 5). Two-way ANOVA followed by Bonferroni multiple comparison test (*p < 0.05, ****p <.0001, vehicle group compared with BCTC; †††*p*<.001, ††††*p* <.0001, exendin 9–39 compared with BCTC). **f**Effects of intraplantar administration of exendin 9–39 (5 and 10 μg) on CAP-induced acute thermal hyperalgesia (left) and mechanical allodynia (right) in mice (mean ± S.E.M., *n* = 5 each). Two-way ANOVA followed by Bonferroni multiple comparison test (*p < 0.05, **p < 0.01, ***p <.001, ****p <.0001, compared with vehicle group; #*p*< 0.05, ##*p* < 0.01, ###*p* <.001, ####*p* <.0001, compared with vehicle + CAP group). ANOVA: analysis of variance, CAP: capsaicin, DRG: dorsal root ganglia, GLP-1: glucagon-like peptide-1, S.E.M: standard error of mean, TRPV1: transient receptor potential vanilloid 1.

We initially sought to examine whether exendin 9–39 could reverse the decrease in pain sensitivity through local intraplantar injection. However, the results of the Hargreaves test indicated that the administration of 10 μg exendin 9–39 decreased heat sensitivity upon intraplantar injection (Fig. 3a).

To determine its effects, we pretreated DRG cultures with 100 nM exendin 9–39. Similar to GLP-1, both CAP-induced TRPV1 inward currents and Ca^2+^ responses were decreased by 85% and 90.6%, respectively (Fig. 3b, c).

To determine the direct effect of exendin 9–39 on the time mice spent licking their hind paw in the CAP-induced spontaneous pain model, intraplantar administration of exendin 9–39 (doses of 5 and 10 μg) was followed by intraplantar administration of CAP (1.6 μg). Exendin 9–39 demonstrated a dose-dependent reduction in paw-licking time, similar to the effect observed with BCTC (0.5 μg) as the control (Fig. 3d). In addition, the paw withdrawal latency (PWL) in the Hargreaves test was significantly lower in the vehicle + CAP than vehicle groups from 30 to 120 min (Fig. 3f). Similarly, intraplantar CAP injection significantly reduced the PWT in the von Frey test from 30 to 120 min compared with that in the vehicle group (Fig. 3f), whereas mechanical allodynia was dose-dependently alleviated in the groups treated with exendin 9–39 (5 or 10 μg) or BCTC (0.5 μg), a widely used TRPV1 inhibitor^39^. These results suggest that the analgesic effects of exendin 9–39 via TRPV1 were similar to those of BCTC.

To determine whether exendin 9–39 treatment causes hyperthermia, which is a typical adverse effect of TRPV1 inhibitors^40^, body temperature was measured following the administration of exendin 9–39. Previous animal studies have shown that intraperitoneal administration of various TRPV1 antagonists results in hyperthermia^41^; therefore, in this study, exendin 9–39 was injected intraperitoneally. As exendin 9–39 showed analgesic effects on the nociceptive behavior of mice at a 10 times higher mass compared with that of BCTC, the same factor (50 mg/kg exendin 9–39 vs. 5 mg/kg BCTC) was used in this experiment.

The rectal temperature in the group treated with exendin 9–39 was similar to that in the vehicle-treated group. By contrast, the rectal temperature in the BCTC-treated group rapidly increased by 0.7–1.5°C and returned to baseline values at 12 min post-administration (Fig. 3e).

#### Exendin 9–39 alleviates inflammatory and neuropathic chronic pain behaviors

**Figure 4:**
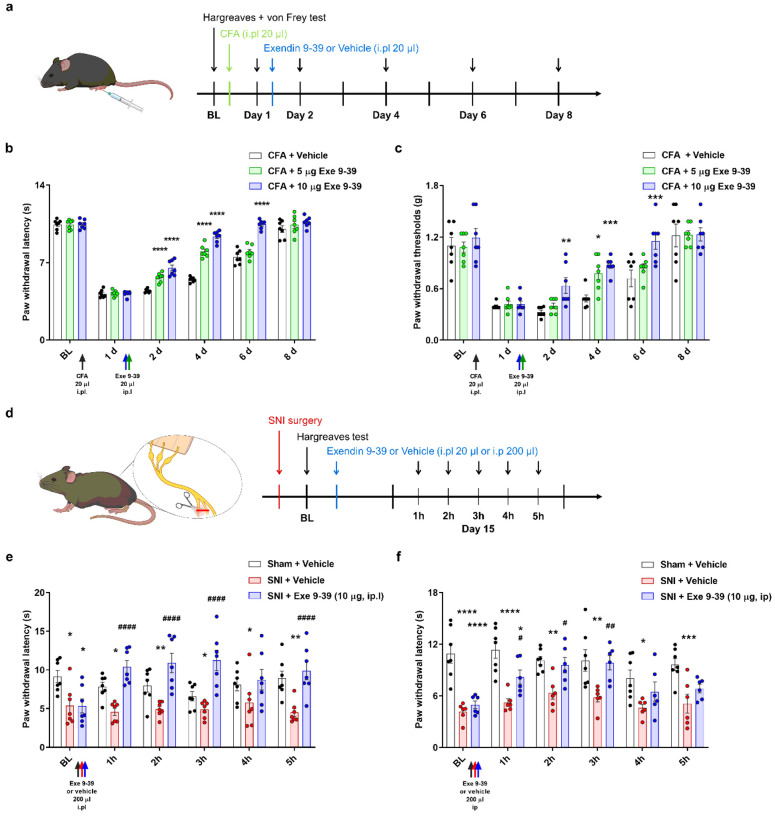
Alleviation of CFA-induced inflammatory and SNI-induced neuropathic pain via exendin 9–39 administration. **a**Schematic illustration and timeline to induce CFA-induced inflammatory chronic pain model in mice. **b** Effects of intraplantar injection of exendin 9–39 (Exe 9–39) (5 and 10 μg) on thermal hyperalgesia in CFA-induced inflammatory chronic pain mouse model using the Hargreaves test (mean ± S.E.M., *n* = 7 each). Two-way ANOVA followed by Bonferroni multiple comparison test (****p<.0001, compared with CFA group). **c** Effects of intraplantar injection of exendin 9–39 (Exe 9–39) (5 and 10 μg) on mechanical allodynia in CFA-induced inflammatory chronic pain mouse model using the von Frey test (mean ± S.E.M., *n*= 7 each). Two-way ANOVA followed by Bonferroni multiple comparison test (*p< 0.05, **p < 0.01, ***p <.001, compared with CFA group). **d** Schematic illustration and timeline to induce SNI-induced neuropathic chronic pain model in mice. **e** Effects of intraplantar injection of exendin 9–39 (Exe 9–39) (10 μg) on thermal hyperalgesia in SNI-induced neuropathic chronic pain mouse model using the Hargreaves test (mean ± S.E.M., *n* = 7 each). Two-way ANOVA followed by Bonferroni multiple comparison test (*p < 0.05, **p < 0.01, compared with the Sham + Vehicle group; ####*p* <.0001, compared with the SNI + Vehicle group). **f** Effects of intraperitoneal injection of exendin 9–39 (Exe 9–39) (10 μg) on thermal hyperalgesia in SNI-induced neuropathic chronic pain mouse model using the Hargreaves test (mean ± S.E.M., *n* = 7 each). Two-way ANOVA followed by Bonferroni multiple comparison test (*p < 0.05, **p < 0.01, ***p <.001, ****p <.0001, compared with the Sham + Vehicle group; #*p* < 0.05, ##*p* < 0.01, compared with the SNI + Vehicle group). ANOVA: analysis of variance, CFA: complete Freund’s adjuvant, SNI: spared nerve injury, GLP-1: glucagon-like peptide-1, S.E.M: standard error of mean,TRPV1: transient receptor potential vanilloid 1.

The analgesic potential of exendin 9–39 was confirmed using an acute pain mice model, followed by an assessment of its effectiveness in the chronic pain model. Considering the documented changes in sensitization and upregulation of TRPV1 in the complete Freund’s adjuvant (CFA)-induced inflammatory pain model, CFA was utilized to induce chronic inflammatory pain in mice^42^. The effects of exendin 9–39 on heat hyperalgesia and mechanical allodynia were assessed using the Hargreaves and von Frey tests, respectively (Fig. 4a). In the Hargreaves test, mice with CFA-induced inflammation displayed delayed recovery from heat hyperalgesia following intraplantar vehicle administration. However, a single intraplantar administration of 5 or 10 μg exendin 9–39 effectively relieved heat hyperalgesia and accelerated the recovery process (Fig. 4b). Similarly, in the von Frey test, mice injected with intraplantar vehicle post-CFA induction displayed prolonged mechanical allodynia, which was significantly reversed by single intraplantar administration of 5 or 10 μg exendin 9–39 (Fig. 4c). Additionally, exendin 9–39 administration mitigated paw swelling resulting from CFA induction (Supplementary Fig. 2).

Furthermore, peripheral nerve injury leads to neuropathic pain, and the spared nerve injury (SNI) model in mice induces persistent heat hyperalgesia^43^. After 14 days of SNI surgery, mice showed heat hyperalgesia compared with those in the sham group with intraplantar vehicle administration (Fig. 4d).

However, SNI-challenged mice when administered intraplantar injections of 10 μg exendin 9–39 showed alleviated heat hyperalgesia starting from 1 h post-administration, peaking at 3 h, compared with those in the vehicle-administered group (Fig. 4e). Similarly, when the same dose of exendin 9–39 was administered intraperitoneally to test systemic analgesic effects, it alleviated heat hyperalgesia starting from 1 h post-administration, peaking at 3 h, compared with the intraperitoneal administration of the vehicle (Fig. 4f). These findings suggest that the analgesic effects of exendin 9–39 extend not only to chronic inflammatory pain but also to neuropathic pain.

**Figure 5:**
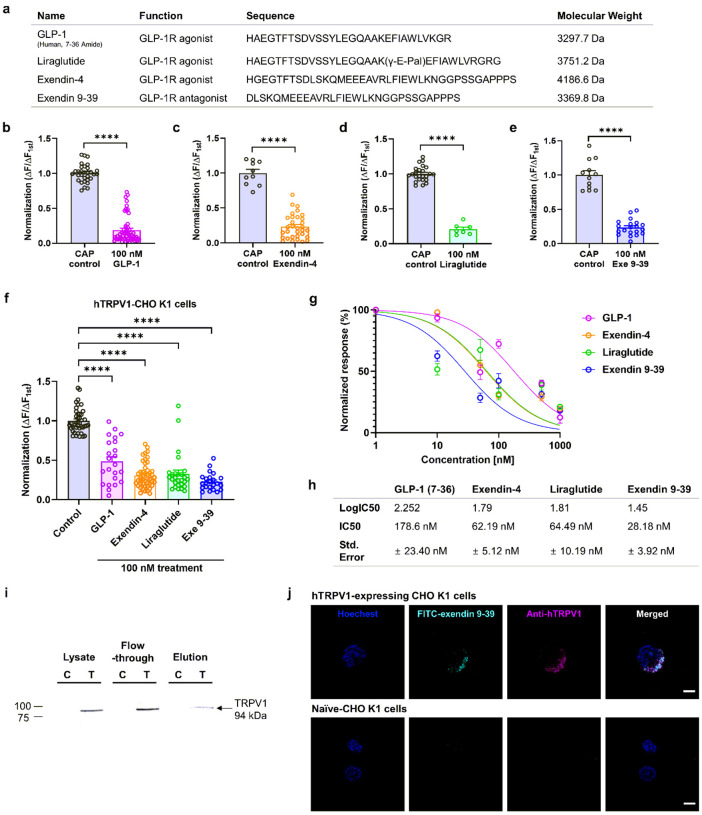
Inhibition of CAP-induced calcium influx via direct interaction with TRPV1 by GLP-1 analogs and exendin 9–39 in HEK293T cells transfected with rat TRPV1 and CHO K1 cells expressing human TRPV1. **a** Sequence alignment, molecular weight, and function of GLP-1 analogs and exendin 9–39. **b–e**Mean normalized calcium influx in HEK293T cells transfected with rat TRPV1. Calcium increases were elicited by CAP (100 nM) and the presence of GLP-1 analogs (100 nM each), GLP-1(7–36) (**b**), exendin-4 (**c**), liraglutide (**d**), or exendin 9–39 (Exe 9–39) (**e**) (mean ± S.E.M.). Two-tailed unpaired *t*-test (****p <.0001, compared with each control CAP). **f** Mean normalized calcium influx in CHO K1 cells expressing human TRPV1. Calcium increases were elicited by CAP (100 nM) in the absence (control CAP) and the presence of GLP-1 analogs (100 nM) or exendin 9–39 (100 nM) (mean ± S.E.M.). One-way ANOVA followed by Dunnett’s multiple comparison test (****p <.0001, compared with control CAP). **g**Curves were fitted using a logistic function for concentration-dependent inhibition of CAP-induced TRPV1 currents by 100 nM of GLP-1, exendin-4, liraglutide, and exendin 9–39. **h** IC50 values of GLP-1, exendin-4, liraglutide, and exendin 9–39 (178.6 ± 23.40 nM, 62.19 ± 5.12 nM, 64.49 ± 10.19 nM, and 28.18 ± 3.92 nM, respectively). **i** Pull-down assay using His-tagged exendin 9–39 and cell lysates from CHO K1 cells expressing human TRPV1 (‘T’) and naïve CHO K1 cells (‘C’). Detection for the interaction with exendin 9–39 and TRPV1 using western blotting with anti-human TRPV1 antibody. **j**Confocal images of FITC-labeled exendin 9–39 in CHO K1 cells expressing human TRPV1 (upper) compared with naïve CHO K1 cells (lower). Overlap of FITC-exendin 9–39 (green), anti-human TRPV1 antibody (red), and Hoechst (blue) in the merged image. Scale bar: 5 μm. ANOVA: analysis of variance, CAP: capsaicin, CHO K1, Chinese hamster ovary, FITC: fluorescein isothiocyanate GLP-1: glucagon-like peptide-1, S.E.M: standard error of mean,TRPV1: transient receptor potential vanilloid 1.

GLP-1-derived peptides regulate rat and human TRPV1 activation independently from GLP-1R expression To verify the potential involvement of GLP-1R in the observed inhibitory effects on TRPV1 channels, we transiently transfected rat TRPV1 into HEK293T cells. GLP-1R expression was not observed in the HEK293T cell line before transfection (Supplementary Fig. 3). CAP-induced TRPV1 calcium responses were again inhibited by pretreatment with GLP-1 analogs or exendin 9–39 (Fig. 5a–e), indicating no interaction with GLP-1R was involved. Next, we estimated and compared the half-maximal inhibitory concentration (IC_50_) values from the concentration-response data using Chinese hamster ovary (CHO K1) cells expressing human TRPV1. The IC_50_ values based on calcium response for GLP-1, liraglutide, exendin-4, and exendin 9–39 were 178.60, 62.19, 64.49, and 28.18 nM, respectively (Fig. 5f–h). To determine the subtype selectivity of exendin 9–39, we tested its effect on TRPA1. Exendin 9–39 did not inhibit or potentiate TRPA1 currents induced by 100 μM allyl isothiocyanate (AITC) (Supplementary Fig. 4a–c), demonstrating that exendin 9–39 is a selective inhibitor of TRPV1. As exendin 9–39 showed the lowest IC_50_ value for inhibiting CAP-activated TRPV1 currents, its molecular mechanism was further analyzed.

### Exendin 9–39 directly binds to the TRPV1 channel

To assess the direct interaction between exendin 9–39 and the TRPV1 channel, we conducted a protein binding assay using His-tagged exendin 9–39, which was able to pull down the TRPV1 channel (Fig. 5i) in the CHO K1 cell line stably expressing human TRPV1. In contrast, the naïve CHO K1 cell line showed no TRPV1 channel binding. We tested the binding of exendin 9–39 to TRPV1 by incubating the two cell lines with fluorescein isothiocyanate (FITC)-labeled exendin 9–39. Immunofluorescence imaging revealed that FITC-labeled exendin 9–39 was bound to TRPV1 on the cell surface in CHO K1 cells expressing human TRPV1 but not in naïve CHO K1 cells (Fig. 5j). Although exendin 9–39 was tagged with FITC, it still blocked the CAP-induced inward currents of TRPV1 channels (Supplementary Fig. 5a, b).

### Exendin 9–39 targets the extracellular side of the TRPV1 channel but does not share the CAP binding site

**Figure 6:**
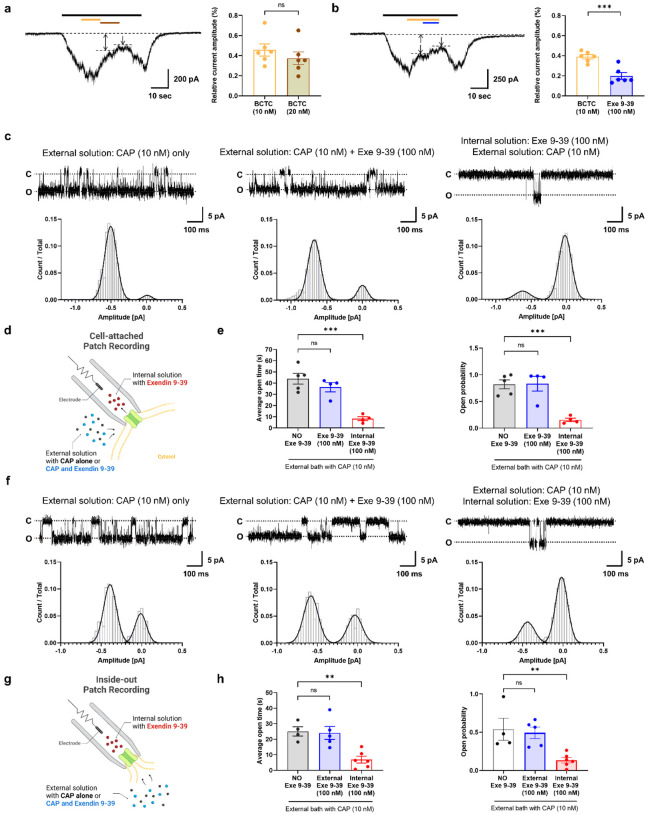
Exendin 9–39 non-competitively inhibits CAP-induced TRPV1 activation by binding to the extracellular side in CHO K1 cells expressing human TRPV1. **a** Currents evoked by 100 nM CAP were partially blocked by sequential application of 10 and 20 nM BCTC (left). The remaining currents between the arrows were CAP-induced currents inhibited by BCTC. Summary analysis of the inward currents reduced by BCTC alone (mean ± S.E.M.). Two-tailed unpaired t-test (ns, not significant). **b** Currents evoked by 100 nM CAP were partially blocked by sequential application of 10 nM BCTC and 100 nM exendin 9–39 (Exe 9–39). The remaining currents between the arrows were CAP-induced currents inhibited by BCTC- or exendin 9–39. Summary of inward currents reduced by BCTC alone and BCTC followed by exendin 9–39 (mean ± S.E.M.). Two-tailed unpaired *t*-test (***p <.001). **c**Representative traces from single-channel recordings of TPRV1 channels in the cell-attached configuration. Cells were held at −60 mV. Exendin 9–39 (100 nM) was delivered either via the external (intracellular) or internal (extracellular) solution in the presence of 10 nM CAP externally. c: closed state; o: open state. **d** Schematic illustration of cell-attached patch recordings. **e** Average single-channel open time (left) and open probability (right) (mean ± S.E.M.). One-way ANOVA followed by Dunnett’s multiple comparison test (***p <.001, compared with no exendin 9–39). **f** Representative traces from single-channel recordings of TPRV1 channels in the inside-out configuration. Cells were held at −60 mV. Exendin 9–39 (100 nM) was delivered either via the external (intracellular) or internal (extracellular) solution in the presence of 10 nM CAP externally. c: closed state; o: open state. **g** Schematic of inside-out patch recordings. **h**Average single-channel open time (left) and open probability (right) (mean ± S.E.M.). One-way ANOVA followed by Dunnett’s multiple comparison test (**p< 0.01, compared with no exendin 9–39). ANOVA: analysis of variance, BCTC: N-(4-tertiarybutylphenyl)-4-(3-cholorphyridin-2-yl) tetrahydropyrazine-1(2H)-carboxamide, CAP: capsaicin, CHO K1, Chinese hamster ovary, S.E.M: standard error of mean,TRPV1: transient receptor potential vanilloid 1.

Next, we examined the molecular target of exendin 9–39 on the TRPV1 channel using a patch-clamp competition assay in CHO K1 cells expressing human TRPV1. BCTC, a highly potent TRPV1 antagonist that competitively interacts with CAP to bind to TRPV1 channels^39^, was used for this assay. BCTC (10 nM) was applied in the presence of 10 nM CAP until saturation, and twice the BCTC concentration (20 nM) was sequentially applied. While 10 nM BCTC blocked the CAP-induced TRPV1 currents, 20 nM BCTC did not significantly potentiate this TRPV1-inhibiting effect (Fig. 6a). In contrast, 100 nM exendin 9–39 application following 10 nM BCTC elicited a significant further inhibition of CAP-evoked TRPV1 inward currents (Fig. 6b). These results indicate that exendin 9–39 does not share the binding site with CAP and inhibits TRPV1 activation by interacting noncompetitively with CAP.

To validate the TRPV1 binding site, we performed single-channel recordings from cells expressing human TRPV1 channels. Cell-attached patch recordings showed that the external bath application of 10 nM CAP and 100 nM exendin 9–39 did not reduce the frequency of single-channel open events. In contrast, when the pipette solution contained 100 nM exendin 9–39, which allowed the interaction of exendin 9–39 with the extracellular surface of TRPV1, single-channel opening events were significantly blocked (Fig. 6c–e). We further conducted inside-out patch-clamp recordings to determine whether bath application of CAP and exendin 9–39 to the intracellular surface of TRPV1 changed single-channel opening events (Fig. 6f–h). The results showed that only if the pipette solution contained 100 nM exendin 9–39, facilitating the exposure of exendin 9–39 to the extracellular surface of TRPV1, were the single-channel openings blocked while CAP was applied to the intracellular side of the channel. In contrast, single-channel opening events were not detected when CAP and exendin 9–39 were administered via the external bath, allowing exposure of CAP and exendin 9–39 to the intracellular surface of TRPV1. Thus, exendin 9–39 binds to the extracellular side of TRPV1 but cannot penetrate the membrane.

### Exendin 9–39 does not affect TRPV1 activation by protons suggesting its mode selectivity

**Figure 7:**
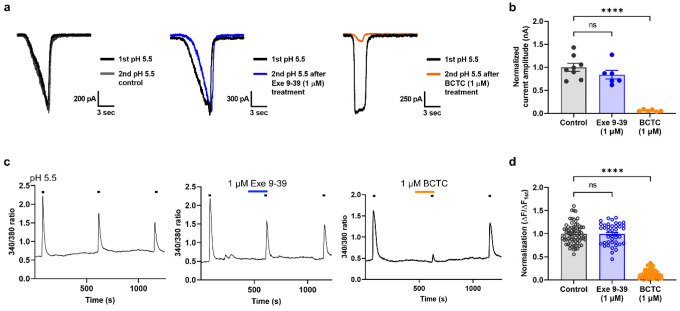
No effect of exendin 9–39 on proton-induced TRPV1 currents and calcium influx in CHO K1 cells expressing human TRPV1. **a** Representative proton-induced inward currents evoked by pH 5.5 under control conditions (blue) or with 1 μM exendin 9–39 (blue) or 1 μM BCTC (orange) pretreatment. **b** Mean normalized current amplitude of TRPV1 currents (nA) after treatment with 1 μM exendin 9–39 or 1 μM BCTC compared with control pH 5.5 (blue) (mean ± S.E.M.). One-way ANOVA followed by Dunnett’s multiple comparison test (ns, not significant; ****p <.0001, compared with control pH 5.5). **c** Representative proton-induced calcium influx by pH 5.5 under control conditions (blue) or with 1 μM exendin 9–39 (blue) or 1 μM BCTC (orange) pretreatment. **d** Mean normalized 340/380 ratios of sequential proton-induced calcium increases (mean ± S.E.M.). One-way ANOVA followed by Dunnett’s multiple comparison test (ns, not significant; ****p <.0001, compared with control pH 5.5). ANOVA: analysis of variance, BCTC: N-(4-tertiarybutylphenyl)-4-(3-cholorphyridin-2-yl) tetrahydropyrazine-1(2H)-carboxamide, CHO K1, Chinese hamster ovary, S.E.M: standard error of mean, TRPV1: transient receptor potential vanilloid 1.

As the inhibitory effect of exendin 9–39 on CAP-induced activation of TRPV1 was confirmed, its effect on proton-induced activation of TRPV1 was also tested using patch-clamp recordings and calcium imaging. BCTC is also a potent antagonist of TRPV1 activated by protons^44^; therefore, it was used as a control antagonist. Unlike exendin 9–39 during CAP-induced TRPV1 activation, 1 μM exendin 9–39 did neither reduce nor potentiate low pH-induced inward currents (Fig. 7a, b) and calcium influx (Fig. 7c, d) in hTRPV1-expressing CHO K1 cells, whereas 1 μM BCTC completely blocked them. The exendin 9–39 concentration used in this experiment was approximately 30 times higher than its IC_50_ value in CAP-induced TRPV1 activation, indicating the mode-selective characteristics of exendin 9–39. This suggests that exendin 9–39 may be less likely to cause adverse effects associated with thermoregulation, such as hyperthermia or hypothermia, as found with previous TRPV1 antagonists.

### Small peptide derived from exendin 9–39 inhibits TRPV1 activation and attenuates CAP-induced spontaneous pain in mice

**Figure 8:**
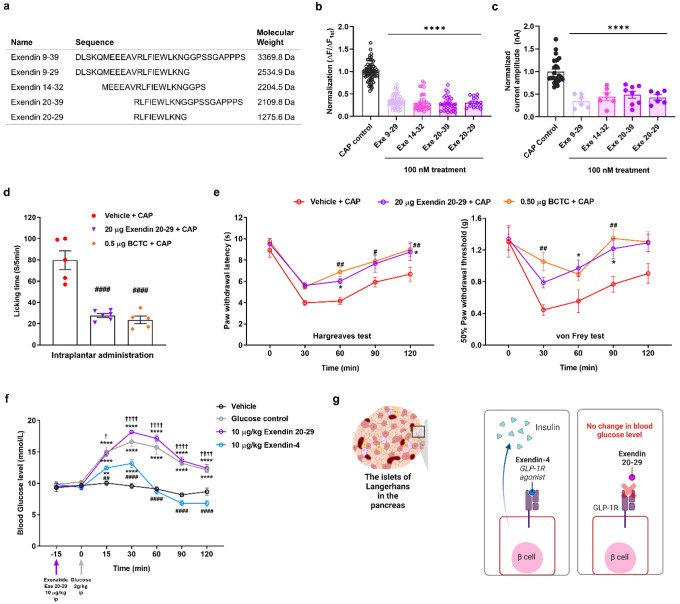
Inhibition of CAP-induced TRPV1 activation by exendin 9–39 fragments and analgesic effects of exendin 20–29 on CAP-induced nociceptive behaviors in mice. **a**Sequence alignment of three truncated small peptides derived from exendin 9–39, their overlapping sequence exendin 20–29, and the molecular weights of these peptides. **b** Mean normalized calcium influx in CHO K1 cells expressing human TRPV1 with control CAP (100 nM) and each of the four truncated small peptides derived from exendin 9–39 (100 nM each) (mean ± S.E.M.). One-way ANOVA followed by Dunnett’s multiple comparison test (****p <.0001, compared with control CAP). **c** Mean normalized currents of sequential CAP-induced currents (mean ± S.E.M.). One-way ANOVA followed by Dunnett’s multiple comparison test (****p <.0001, compared with control CAP). **d**Effects of intraplantar administration of exendin 20–29 (20 μg) and BCTC (0.5 μg) on CAP (1.6 μg)-induced acute licking time (mean ± S.E.M., *n* = 6). One-way ANOVA followed by Dunnett’s multiple comparison test (##*p* < 0.01, compared with vehicle + CAP group). **e** Effects of intraplantar administration of exendin 20–29 (20 μg) on CAP-induced acute thermal hyperalgesia (left) and mechanical allodynia (right) in mice. Two-way ANOVA followed by Bonferroni multiple comparison test (*p < 0.05, vehicle + CAP group compared with exendin 20–29 [20 μg]; #*p* < 0.05, ##*p*< 0.01, vehicle + CAP group compared with BCTC [0.5 μg] + CAP). **f**Effects of intraperitoneal administration of 10 μg/kg exendin 20–29 or 10 μg/kg exendin-4 on blood glucose levels following administration of 2 g/kg glucose (mean ± S.E.M., *n* = 5). Two-way ANOVA followed by Bonferroni multiple comparison test (**p < 0.01, ****p <.0001, compared with vehicle group; ##*p* < 0.01, ####*p* <.0001, compared with glucose control; †*p* < 0.05, ††††*p* <.0001, compared with exendin-4). **g** Schematic illustration of the potential mechanism by which exendin 20–29 does not disrupt the regulation of blood glucose levels in the pancreas, where GLP-1 receptors are present. ANOVA: analysis of variance, BCTC: N-(4-tertiarybutylphenyl)-4-(3-cholorphyridin-2-yl) tetrahydropyrazine-1(2H)-carboxamide, CAP: capsaicin, CHO K1, Chinese hamster ovary, GLP-1: glucagon-like peptide-1, S.E.M: standard error of mean,TRPV1: transient receptor potential vanilloid 1.

To verify the lack of GLP-1R involvement in the observed exendin 9–39 effects, we roughly fragmentized exendin 9–39 into three small peptides, i.e., exendin 9–29, 14–32, and 20–39 (Fig. 8a), all of which reduced CAP-induced inward currents (Fig. 8b) and calcium responses (Fig. 8c). We, therefore, assumed that the overlapping peptide sequence exendin 20–29 plays a key role in the inhibition of CAP-induced TRPV1 activation. As hypothesized, exendin 20–29 showed similar inhibitory effects (Fig. 8b, c). These results suggest exendin 20–29 as a potential TRPV1 blocker derived from exendin 9–39.

The analgesic effects of exendin 20–29 were then examined in the same manner as those of exendin 9–39. Intraplantar administration of exendin 20–29 (20 μg) also reduced paw-licking time (Fig. 8d). Additional intraplantar injection of exendin 20–29 (20 μg) significantly alleviated pain from 60 to 120 min (Fig. 8e) as manifested in the increased PWL in the Hargreaves test, compared with intraplantar administration of CAP alone (1.6 μg). This alleviation was also observed in the group with additional BCTC (0.5 μg) treatment, and no significant differences between the 20 μg exendin 20–29 + CAP and 0.5 μg BCTC + CAP groups were found. Similarly, intraplantar exendin 20–29 (20 μg) injection significantly increased the PWT in the von Frey test from 30 to 90 min, compared to CAP (1.6 μg) administration alone (Fig. 8e). However, no significant differences were found between the 20 μg exendin 20–29 + CAP and 0.5 μg BCTC + CAP groups. We also determined whether exendin 20–29 modulates proton-induced TRPV1 activation using patch-clamp recordings and calcium imaging. When 1 μM exendin 20–29 was applied, no significant effect was found in contrast to the 92% inhibition elicited by 1 μM BCTC (Supplementary Fig. 6a–c). Collectively, these data reveal that exendin 20–29 acts as a key sequence in inducing analgesia in CAP-elicited nociceptive behaviors in mice, without causing hyperthermia, and this analgesic effect is mediated by exendin 20–29 directly inhibiting CAP-induced TRPV1 activation, but not proton-induced TRPV1 activation.

To determine the potential effects of exendin 20–29 on GLP-1R and glucose regulation, we performed an acute intraperitoneal glucose tolerance test (IPGTT) in 8-week-old mice. As the GLP-1 analog exendin-4 (10 μg/kg) lowers blood glucose levels^45^, the same amount (10 μg/kg) of exendin 20–29 was administered intraperitoneally. After 15 min of exendin 20–29 or exendin-4 administration, a glucose solution (2 g/kg) was intraperitoneally injected (at 0 min; Fig. 8f). In contrast to exendin-4, which significantly lowered the blood glucose level from 15 to 120 min, exendin 20–29 and control (without pretreatment) did not change the glucose level during the experiment (Fig. 8f). This result indicates that exendin 20–29 is less likely to interact with GLP-1R (Fig. 8g). Moreover, if the exendin 20–29 mass effective for pain relief (20 μg) was intraperitoneally injected in wild-type mice without glucose treatment, changes in blood glucose levels were not detected. This indicates that exendin 20–29 does not activate GLP-1R (Supplementary Fig. 7).

#### Exendin 20–29 alleviates inflammatory and neuropathic chronic pain behaviors

Exendin 20–29 was further assessed for its efficacy in alleviating inflammatory and neuropathic chronic pain behaviors. In CFA-induced chronic inflammatory pain model, mice with CFA-induced inflammation showed prolonged heat hyperalgesia following intraplantar vehicle administration, while a single intraplantar administration of 20 or 50 μg exendin 20–29 effectively alleviated heat, with complete recovery observed by day 8 (Supplementary Fig. 8a). Similarly, in the von Frey test, mice injected with intraplantar vehicle post-CFA induction displayed slow recovery of mechanical allodynia. Nevertheless, a single intraplantar administration of 20 or 50 μg exendin 20–29 alleviated mechanical allodynia (Supplementary Fig. 8b). Additionally, administration of exendin 20–29 resulted in a reduction in paw swelling induced by CFA (Supplementary Fig. 8c). Moreover, in the SNI-induced chronic neuropathic pain model, mice challenged with SNI and administered intraperitoneal injections of 50 μg exendin 20–29 exhibited alleviated heat hyperalgesia, with effects observed as early as 1 hour post-administration and peaking at 2 hours, compared to the vehicle-administered group (Supplementary Fig. 8d). These findings highlight the therapeutic potential of exendin 20–29 as an analgesic agent for chronic pain management.

## DISCUSSION

Our study has identified a novel function for GLP-1-derived peptides in providing pain relief. We specifically found that exendin 20–29 inhibits TRPV1 activity in sensory neurons in a direct and mode-specific manner, reducing pain behaviors without noticeable adverse effects. This discovery is particularly relevant in the context of the ingestion analgesia phenomenon, which relates to the suppression of noxious heat-induced withdrawal behaviors in rats during the consumption of chocolate or sweet liquids, contrasting with salt ingestion^3–7^. Our primary aim was to dissect the role of the incretin peptide hormone, GLP-1, in this context by systemically administering GLP-1 and glucose via intraperitoneal injection. Our findings demonstrated that both natural and synthetic GLP-1 administrations altered heat sensitivity and that local administration of GLP-1 notably reduced heat sensitivity and CAP-induced spontaneous pain behavior. This suggests that GLP-1 and its metabolites might modulate TRPV1 activity, which plays a pivotal role in heat pain perception^19,46^.

Moreover, our study highlighted that GLP-1 analogs, including GLP-1, liraglutide, and exendin-4, along with the GLP-1 antagonist exendin 9–39, which exhibits 53% sequence homology with native GLP-1^38^, effectively inhibited CAP-induced TRPV1 activation in mouse DRG neurons. Unlike BCTC, exendin 9–39 also mitigated both thermal and mechanical nociceptive behaviors in mice without inducing hyperthermia, marking it as a viable analgesic without the common adverse effects associated with existing pain management strategies, such as opioids and traditional TRPV1 antagonists^32,41,47–49^. Thereby, our study indicated that GLP-1-derived peptides offer effective pain relief without these significant adverse effects, suggesting their potential as a safer alternative for chronic pain management.

Previous studies have shown that GLP-1 analogs can prevent or improve diabetic neuropathy in animal models^50–53^. In addition to their neuroprotective role, GLP-1 analogs have also been studied for their influence on spinal excitatory synaptic transmission under pathophysiological conditions such as neuropathic pain. However, previous studies have not investigated GLP-1 analogs and exendin 9–39 for pain transmission at peripheral sites, especially in small-sized nociceptive DRG neurons, where the TRPV1 channel is crucial in chronic pain-inducing mechanical allodynia and thermal hyperalgesia^54–57^.

To identify the mechanism behind the analgesic effects of GLP-1 analogs and exendin 9–39, we utilized a cell line lacking GLP-1R to transfect the rat TRPV1 plasmid. Both GLP-1 analogs and exendin 9–39 were found to reduce CAP-induced inward currents and calcium influx through the TRPV1 channel. Determination of the IC50 values for these compounds in CHO K1 cells expressing human TRPV1 revealed that exendin 9–39 exhibited the lowest IC_50_ value for inhibiting CAP-induced TRPV1 activation. These findings suggest exendin 9–39 directly interacts with TRPV1, independently of GLP-1R, to inhibit its activation. This interaction was further corroborated by pull-down assays and confocal imaging, indicating a direct binding between exendin 9–39 and TRPV1 channels. Our electrophysiological analyses revealed that exendin 9–39 likely binds to the extracellular side of TRPV1, offering selective and potent antagonism for pain relief. This finding is particularly significant as CAP is known to bind to the transmembrane domains of TRPV1 channels^56^, suggesting exendin 9–39 as a selective and potent antagonist for pain relief without competitively displacing CAP at its binding site.

Although the intraperitoneal administration of exendin 9–39 did not cause the adverse effect of hyperthermia, a critical aspect of developing TRPV1 antagonists is minimizing interference with TRPV1 activation by protons, and hence, identifying mode-selective antagonists^32^. Unlike its influence on CAP-induced TRPV1 activation, exendin 9–39 did not block or potentiate proton-induced channel activity, even at a concentration approximately 30 times higher than its IC_50_ value inhibiting CAP-induced TRPV1 activity, indicating mode-specific inhibition of exendin 9–39.

Critical to our analysis was the examination of the half-maximal effective concentration values for GLP-1 analogs to activate GLP-1R, which are known to be at picomolar levels^58^. To exclude any potential effects of exendin 9–39 on insulin secretion via GLP-1R interaction, we analyzed key protein sequences involved in TRPV1 inhibition. Three distinct fragments of exendin 9–39 were found to reduce CAP-induced inward currents and calcium influx through TRPV1. Their common sequence, exendin 20–29, specifically inhibited CAP-induced TRPV1 activation without affecting proton-induced activation, and similarly alleviated CAP-induced nociceptive behaviors. To ensure that exendin 20–29 does not interact with GLP-1R, we utilized an acute IPGTT. Unlike exendin-4, exendin 20–29 did not affect blood glucose levels, supporting its lack of GLP-1R interaction. This was further validated by the absence of blood glucose changes in wild-type mice following administration of exendin 20–29 at effective doses, contrasting with the hypoglycemic effect observed with exendin-4.

While our results highlight exendin 20–29 as a promising candidate for developing safer peptide analgesics targeting TRPV1, the translation of these findings into clinical practice necessitates further investigation. This includes a thorough examination of the pharmacokinetics and dynamics in human physiology as compared to the animal models used in our study. Given the increasing interest in peptide drugs for their low production costs, high potency, selectivity, and biological stability, our research underscores the potential of finding a peptide analgesic that precisely targets and modulates TRPV1 without causing adverse effects. This represents a promising strategy in the quest for safer and more effective pain management solutions, signaling a potential shift towards innovative analgesic strategies that prioritize both efficacy and patient safety.
